# Mesenchymal stem/stromal cell-based therapies for severe viral pneumonia: therapeutic potential and challenges

**DOI:** 10.1186/s40635-021-00424-5

**Published:** 2021-12-31

**Authors:** C. H. Masterson, A. Ceccato, A. Artigas, C. dos Santos, P. R. Rocco, S. Rolandsson Enes, D. J. Weiss, D. McAuley, M. A. Matthay, K. English, G. F. Curley, J. G. Laffey

**Affiliations:** 1 Anaesthesia, School of Medicine, National University of Ireland, Galway, Ireland; 2 Regenerative Medicine Institute, National University of Ireland, Galway, Ireland; 3 Intensive Care Unit, Hospital Universitari Sagrat Cor, Barcelona, Spain; 4 CIBER de Enfermedades Respiratorias (CIBERES), Sabbadell, Spain; 5 Critical Center, Corporacion Sanitaria Universitaria Parc Tauli, Autonomous University of Barcelona, Sabadell, Spain; 6 Keenan Center for Biomedical Research, St. Michael’s Hospital, Bond St, Toronto, Canada; 7 Interdepartmental Division of Critical Care Medicine and Institutes of Medical Sciences, University of Toronto, Toronto, Canada; 8 Laboratory of Pulmonary Investigation, Carlos Chagas Filho Institute of Biophysics, Federal University of Rio de Janeiro, Rio de Janeiro, Brazil; 9 National Institute of Science and Technology for Regenerative Medicine, Rio de Janeiro, Brazil; 10 Department of Experimental Medical Science, Faculty of Medicine, Lund University, Lund, Sweden; 11 Department of Medicine, University of Vermont College of Medicine, Burlington, VT 05405 USA; 12 Regional Intensive Care Unit, Royal Victoria Hospital, Belfast, UK; 13 Wellcome-Wolfson Institute for Experimental Medicine, Queen’s University Belfast, Belfast, UK; 14 Department of Medicine and Anesthesia, University of California, San Francisco, CA USA; 15 Cardiovascular Research Institute, University of California, San Francisco, CA USA; 16 Department of Biology, Maynooth University, Maynooth, Co. Kildare Ireland; 17 Kathleen Lonsdale Institute for Human Health Research, Maynooth University, Maynooth, Co. Kildare Ireland; 18 Anaesthesia, School of Medicine, Royal College of Surgeons in Ireland, Dublin 9, Ireland; 19 Department of Anaesthesia and Intensive Care Medicine, Galway University Hospitals, Saolta University Hospital Group, Galway, Ireland

**Keywords:** Cell therapy, Mesenchymal stem cells, Pneumonia, Coronavirus, Influenza, Acute respiratory distress syndrome, Sepsis, Critical illness, Pandemic, Acute hypoxic respiratory failure

## Abstract

Severe viral pneumonia is a significant cause of morbidity and mortality globally, whether due to outbreaks of endemic viruses, periodic viral epidemics, or the rarer but devastating global viral pandemics. While limited anti-viral therapies exist, there is a paucity of direct therapies to directly attenuate viral pneumonia-induced lung injury, and management therefore remains largely supportive. Mesenchymal stromal/stem cells (MSCs) are receiving considerable attention as a cytotherapeutic for viral pneumonia. Several properties of MSCs position them as a promising therapeutic strategy for viral pneumonia-induced lung injury as demonstrated in pre-clinical studies in relevant models. More recently, early phase clinical studies have demonstrated a reassuring safety profile of these cells. These investigations have taken on an added importance and urgency during the COVID-19 pandemic, with multiple trials in progress across the globe. In parallel with clinical translation, strategies are being investigated to enhance the therapeutic potential of these cells in vivo, with different MSC tissue sources, specific cellular products including cell-free options, and strategies to ‘licence’ or ‘pre-activate’ these cells, all being explored. This review will assess the therapeutic potential of MSC-based therapies for severe viral pneumonia. It will describe the aetiology and epidemiology of severe viral pneumonia, describe current therapeutic approaches, and examine the data suggesting therapeutic potential of MSCs for severe viral pneumonia in pre-clinical and clinical studies. The challenges and opportunities for MSC-based therapies will then be considered.

**Supplementary Information:**

The online version contains supplementary material available at 10.1186/s40635-021-00424-5.

## Introduction

Severe viral pneumonia is a significant cause of morbidity and mortality globally – one need look no further than the current COVID-19 pandemic to appreciate this. Outside of pandemic settings, viral-induced pneumonias remain common, both due to periodic epidemics, and from seasonal outbreaks of endemic disease. While anti-viral therapies exist, there is a paucity of direct therapies to directly attenuate viral pneumonia-induced lung injury. Management therefore remains largely supportive, including assistance with gas exchange and management of complications and super-infections.

Mesenchymal stromal/stem cells (MSCs) can be isolated from several tissues, such as bone marrow, adipose tissue, and umbilical cord[1, 2], and are receiving considerable attention as a cytotherapeutic for viral pneumonia. Several properties of MSCs position them as a promising therapeutic strategy for viral pneumonia-induced lung injury. Early phase clinical studies have demonstrated a reassuring safety profile of these cells when administered to healthy and ill patients. In addition, strategies are being investigated to enhance the therapeutic potential of these cells in vivo, with different tissue sources, cellular products (cell-free options), and methods of ‘licencing’ or ‘pre-activation’ of these cells, all being explored.

The aim of this review is to assess the therapeutic potential of MSC-based therapies for severe viral pneumonia of different aetiologies. It will describe the aetiology and epidemiology of severe viral pneumonia, describe current therapeutic approaches, and examine the data suggesting therapeutic potential of MSCs for severe viral pneumonia in pre-clinical and clinical studies. The challenges and opportunities for MSC-based therapies will then be considered.

## Aetiology and epidemiology of severe viral pneumonia

Viruses are an increasingly frequent cause of pneumonia [3] in the general population. Before the COVID-19 pandemic, rhinovirus (23.6%), parainfluenza (20.8%), metapneumovirus (18.1%), influenza (16.7%) and respiratory syncytial virus (13.9%) were the main viral causes of severe pneumonia [4] (Table 1).

**Table 1:** Viral causes of severe pneumonia

Most common viruses	Less common viruses
Rhinovirus	Adenovirus
Parainfluenza virus	Varicella-Zoster virus
Metapneumovirus	Hanta virus
Influenza	
Respiratory syncytial virus	
Coronavirus	

Influenza Virus A and B are common causes of severe pneumonia mainly in the fall or winter seasons. In the USA, it is estimated that influenza caused 38,000,000 cases in 2019–2020 season and 22,000 deaths [5]. Worldwide it is estimated that influenza causes 4–9 deaths per 100,000 individuals annually [6]. Over 10% of influenza patients require ICU admission. Severe disease develops mainly in patients with increased age and underlying conditions [7–9], due to isolated influenza infection or by a secondary bacterial co-infection, frequently with *Staphylococcus aureus* (often methicillin-resistant) or *Streptococcus pneumoniae* [10].

Influenza can cause pandemic disease. In 1918 Spanish flu caused 20–100 million deaths worldwide [11]. In 2009, the Swine-Origin Influenza A (H1N1) virus pandemic started in Mexico [12] and rapidly spread worldwide. Influenza A H1N1 caused severe disease with pneumonia and acute respiratory distress syndrome (ARDS) and extrapulmonary disease. Younger patients, female, immunosuppression, obesity and pregnancy were risk factors for severe disease [13]. ICU mortality in H1N1-infected patients was approximately 40% [12]. Avian-origin Influenza has also been reported to cause human disease in isolated cases, but to date widespread human dissemination has not occurred [14].

Since 2000, several novel coronaviruses (CoVs) causing severe pneumonia have emerged. In 2003, severe acute respiratory syndrome (SARS), caused by the novel SARS-CoV emerged in the southeast of China, spreading throughout southeast Asia. More than 8000 patients were diagnosed, with 774 deaths reported in 26 countries during 2003 [15]. Spread was rapid, with nosocomial outbreaks affecting health care workers a feature [16]. In 2012, Middle-Eastern respiratory syndrome (MERS), caused by MERS-CoV, was first isolated in Saudi Arabia [17]. Since 2012 MERS outbreaks have been reported in 27 countries and 35% of patients died [18].

The SARS-CoV2 virus, which emerged in Wuhan China in late 2019, and was declared a pandemic by the WHO in March 2020, has infected over 250 million people, with over 4.5 million deaths reported (likely underestimated) to date [19]. Older age, immunosuppression, obesity, diabetes, hypertension, COPD, higher renal and cardiovascular SOFA score components, lower PaO_2_/FiO_2_ ratio, neutrophilia, higher LDH, D-dimer and a shorter time between first symptoms and ICU admission are associated with mortality [20–22]. Patients requiring invasive mechanical ventilation have a 45% mortality [23], with up to 80% of patients over 80 years of age dying [24]. Long-term morbidity after COVID-19 (‘Long COVID’), with severe alteration on chest-CT, decreased pulmonary functions and quality of life, is widely reported [25, 26].

## Mechanisms of lung injury in viral pneumonia

The alveolar–capillary unit is disrupted during severe viral pneumonia [27], with upregulation of pro-inflammatory mediators, infiltration of neutrophils and monocyte–macrophages into the vascular and alveolar compartments increasing capillary endothelial permeability, resulting in ARDS [27]. Notable differences  exist in the biologic profile of COVID-19 relative to classical ARDS, with lower expression of interferons (IFNs) and an increase in thrombotic mediators [28]. An understanding of the viral replication cycle, and the associated host response, highlights the unique pathophysiology of viral pneumonia.

Influenza viruses may infect a variety of lung cells, including ciliated epithelial cells, type I and II alveolar cells, and immune cells [29]. Virus tropism is due to the ability of influenza viruses to bind different isoforms of sialic acid present on host cells via hyaluronidase. The higher virulence of some influenza subtypes (e.g. the “avian” H5N1) may also be related to their greater affinity for the sialyl-galactosyl residues present in the distal respiratory tract, thus leading to more severe lung involvement [29].

The coronaviruses (SARS-CoV, MERS-CoV, and SARS-CoV-2) exploit distinct receptors to enter host cells. MERS-CoV binds to the dipeptidyl peptidase-4 (DPP4), a surface protein mainly expressed on alveolar macrophages and, to a lesser extent, on alveolar epithelial cells and T cells [30, 31]. This marked tropism for immune rather than lung cells is the basis of MERS-CoV immunopathogenesis. SARS-CoV-2 has tropism for ciliated airway epithelial cells and type II alveolar epithelial cells, which is conferred by its dependence on both the human angiotensin converting enzyme-2 (ACE2) receptor, as well as the host transmembrane serine protease-2 (TMPRSS2) for S-protein cleavage and subsequent activation [32]. An important, receptor-mediated pathogenic process derives from the dysregulation of the renin–angiotensin system (RAS) induced by SARS-CoV and SARS-CoV-2. By binding to ACE2, they induce both its internalization and shedding through ADAM17 activation; a reduced ACE2 activity results in increased vascular permeability, enhanced lung oedema, and worsening lung damage and the pro-inflammatory response [33, 34].

Upon viral sensing, pattern recognition receptors (PRRs), such as retinoic acid-inducible gene (RIG-I)-like receptors (RLRs) and nucleotide-binding oligomerization domain (NOD)-like receptors (NLR), such as NLRP3, activate signalling pathways that trigger the release of type I and III IFNs, as well as pro-inflammatory mediators, including cytokines, chemokines, and antimicrobial peptides, that assist in the prevention and clearance of respiratory viral infections [35]. The lung injury in the case of primary viral pneumonia is caused, in part, from the overproduction of inflammatory cytokines resulting from virus replication in lung cells [36]. Early work performed to characterize the host immune response of COVID-19 suggested an immune signature consisting of elevated serum cytokines [particularly Interleukin (IL)-1β, IL-6 and tumour necrosis factor (TNF)-α], impaired interferon responses, and peripheral lymphopenia as markers of severe disease; other associated inflammatory serum markers include elevated levels of ferritin, lactate dehydrogenase, d-dimer, C-reactive protein, and coagulation factors [36, 37]. The pro-inflammatory immune signature of SARS-CoV-2 has been likened to macrophage-activation syndrome (MAS), a life-threatening clinical entity observed in autoimmune diseases and mimicked in many viral infections, including influenza [38, 39]. However, reported plasma IL-6 levels in COVID-19 patients appear to be significantly lower on average (10- to 40-fold) when compared with those reported in other non-COVID-19 ARDS cohorts that display signs of a cytokine storm [40]. It is important to note that IL-6 has several important anti-inflammatory as well as anti-viral functions [41]. Recent findings also suggest that in addition to an increase in pro-inflammatory mediator production there may also be disruptions in specific resolution pathways in patients with COVID-19 [42].

Coronavirus replication is generally associated with a delayed and dramatically reduced IFN induction in most cell types, and COVID-19 severity correlates with the degree of impairment [43, 44]. The ability to evade the innate immune response seems to be the highest for SARS-CoV-2, followed by SARS-CoV and MERS-CoV and, generally, human endemic CoVs are worse inhibitors than epidemic and pandemic viruses [45]. In the case of SARS-CoV, this leads to the dysregulated activation of the inflammatory monocyte–macrophage response, in turn causing vascular leakage and impaired B- and T-cell activation [46].

Data from patients who died from COVID-19 suggest that SARS-CoV-2 infects endothelial cells to cause inflammation (endothelialitis) [47]. This observed endothelialitis supports the hypothesis that SARS-CoV-2 has tropism for vascular endothelial cells, which express the ACE receptor [48]. Indeed, viral cytotoxicity is likely contributing to the pathogenesis of severe COVID-19, since post-mortem detection of replicating virus is common [49]. Direct endothelial damage could also explain the multi-system organ failure and hypercoagulable state associated with severe COVID-19, since local pulmonary endothelialitis could result in activation of the coagulation cascade and exuberant production of endothelium-derived pro-inflammatory cytokines [50].

## Current therapies for viral pneumonia

Approved anti-viral medications with activity against influenza viruses [51] are used as prophylactic adjuncts to the influenza vaccine to control the infection and/or as treatment to reduce symptoms from influenza (Table 2). Antiviral treatment is recommended as early as possible for any patient with confirmed or suspected influenza who are hospitalized, have severe, complicated, or progressive illness; or are at higher risk for influenza complications [51]. Antivirals may also be considered for healthy, symptomatic outpatients not at high risk for influenza complications, who are diagnosed with confirmed or suspected influenza, based on clinical judgment, if treatment can be initiated within 48 h of illness onset.

**Table 2:** Antiviral medications approved and recommended for treatment and chemoprophylaxis of influenza

Antiviral agent (trade name)	Activity against virus	Use	Safety and efficacy	References
Oseltamivir (Tamiflu®)	Influenza A and B	Treatment	Accelerates time to clinical symptom alleviation, reduces risk of lower respiratory tract complications, and admittance to hospital	[139], [140], [141], [142]
		Chemoprophylaxis	Modest evidence regarding whether treatment changes the risk of hospitalization or death in high risk populations	[141], [142]
Zanamivir (Relenza®)	Influenza A and B	Treatment	Decreases the risk of becoming symptomatic	[141], [143], [144], [145]
		Chemoprophylaxis		
Peramivir (Rapivab®)	Influenza A and B	Treatment	Reduces the time to alleviation of influenza symptoms	[146], [147]
		Chemoprophylaxis		
Baloxavir (Xofluza®)	Influenza A and B	Treatment	Effective in alleviating influenza symptoms and reducing the viral load 1 day after initiation	[148], [149], [150], [151], [57], [152]
		Chemoprophylaxis		
Laninamivir (Inavir®)	Influenza A and B	Treatment	Inhibited the NA activities, reduces duration of symptoms	[153], [154], [155], [56], [156], [55]
		Chemoprophylaxis		

Neuraminidase inhibitors (oseltamivir, zanamivir, and peramivir) block enzymes that cleave sialic acid groups from glycoproteins, effectively preventing release of viral particles—virions—from the host cell [52]. These have activity against both Influenza A and B viruses, and are approved by the U.S. Food and Drug Administration (FDA) and by the European Center for Disease Prevention and Control [53]. Baloxavir is a cap-dependent endonuclease inhibitor that interferes with viral RNA transcription and blocks virus replication [54] and has shown significant promise in a multicenter randomized clinical trial of post-exposure prophylaxis [55–57]. The adamantanes (amantadine, rimantadine) are antivirals that target the Influenza A virus M2 ion channel protein [58]. However, high levels of resistance (> 99%) to adamantanes among circulating Influenza A (H3N2) and influenza A(H1N1)pdm09 (“2009 H1N1”) viruses preclude their recommendation for anti-viral treatment or chemoprophylaxis of currently circulating Influenza A viruses (for detailed discussion see [59]).

Multiple compounds are currently at various stages of investigation for influenza treatment [60–62]. The Influenza Therapeutics Program [63] is a major focus of BARDA (The Biomedical Advanced Research and Development Authority) [64]. Prior to the pandemic, the International Society for Influenza and other Respiratory Virus Diseases held its 6th Antiviral Group (isirvAVG) conference to review emerging therapeutics towards seasonal and pandemic influenza, respiratory syncytial virus, coronaviruses including MERS-CoV and SARS-CoV, human rhinovirus, and other respiratory viruses [65]. Multiple additional compounds are under investigation for the treatment of non-coronavirus, lower respiratory tract infections (Additional file 1: Table S1). In addition to potential toxicity and the rapid development of resistance, the limitation of anti-viral-directed therapy is that it obviates management of the response of the host to infection—a fundamental determinant of outcome in the development of ARDS [66–68].

In summary, while specific anti-viral therapies exist, they must be used early in the disease course to be effective. Their limited efficacy later in the infection process means that the cornerstone of management remains supportive care with respiratory support (oxygen, ventilatory assistance), rest, antipyretics, analgesics, nutrition, and close observation [65]. New therapies to directly attenuate viral-induced pneumonia and lung injury are a priority.

## MSCs—therapeutic potential for viral pneumonia

**Fig. 1:**
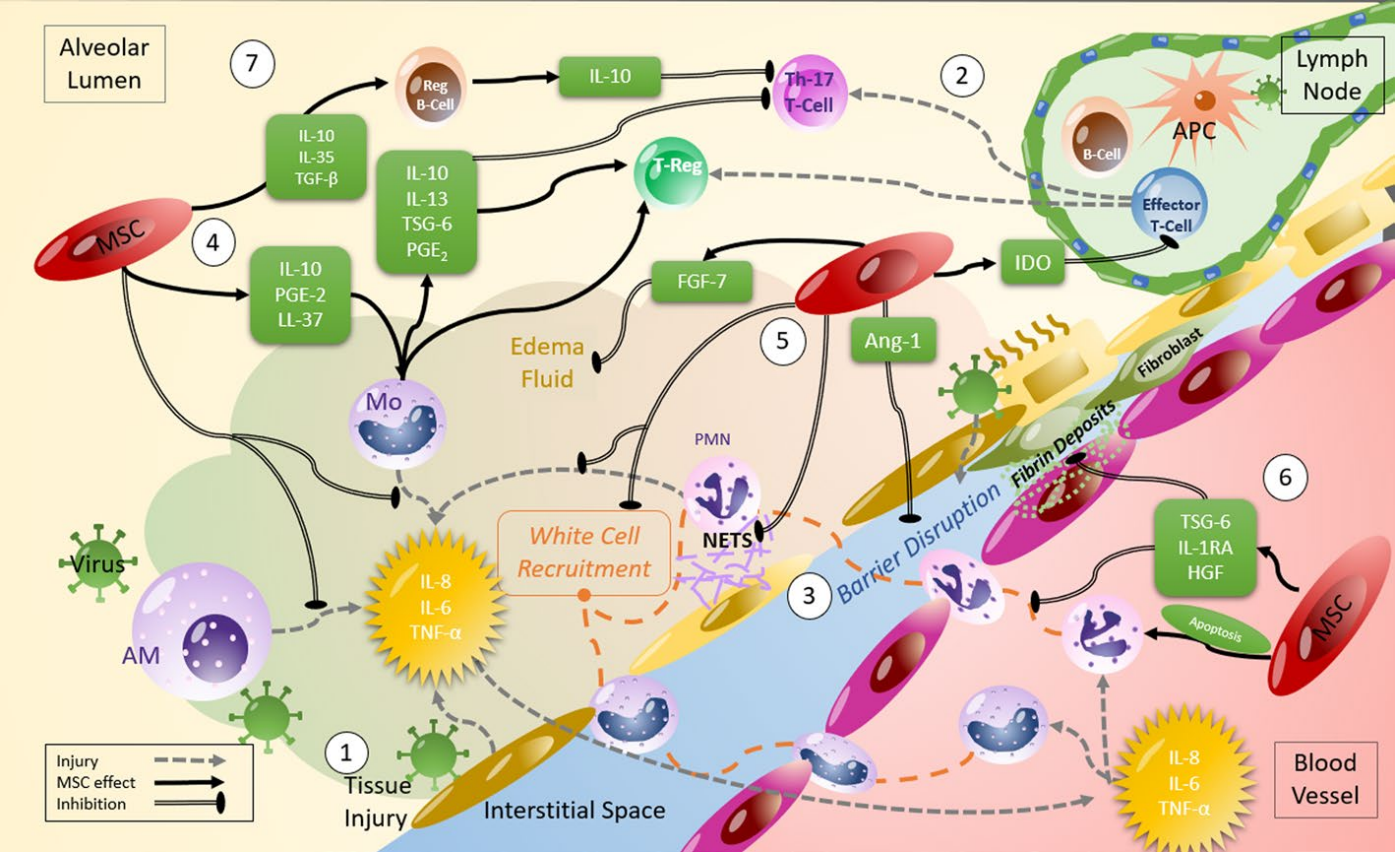
Mechanisms of action of MSCs which can counteract viral infection. (1) Viral infection leads to tissue damage at the delicate blood-air barrier in the lung. The release of inflammatory cytokines initiates further tissue damage with (2) inflammatory T-cell proliferation and differentiation to Th-1 and Th-17s, (3) inflammatory white cell recruitment from the blood and tissues leading to further inflammation, creation of neutrophil NETS, fibroblast differentiation, oedema fluid accumulation and significant barrier disruption. MSCs have been demonstrated to act on several of the injurious processes that occur in infection such as (4) Release of cytokines and chemokines which promote anti-inflammatory innate and adaptive cell phenotypes, (5) release of factors which prevent the formation of NETS, reduce barrier disruption, and (6) prevent fibroblast differentiation and promote PMN apoptosis. (7) MSC IL-10 production and production from anti-inflammatory monocytes induces regulatory B and T cells and promotes tissue protection and repair, and MSC IDO production regulates inflammatory T-cell proliferation

MSCs have been proposed as a promising therapeutic strategy in viral pneumonia because they possess immunomodulatory, anti-microbial, and pro-resolution properties (Fig. 1) [2]. MSCs can be easily sourced from various tissue types, and while the MSC optimal tissue source remains unclear, bone marrow (BM) and umbilical cord (UC) derived MSCs may be more effective than adipose tissue derived MSCs in pre-clinical acute lung injury models [69].

### MSC administration kinetics

Following systemic administration, MSCs are initially trapped in the lungs and subsequently ‘home’ and are retained in injured or inflamed areas [70]. Tracking studies utilizing radiolabelled or fluorescently labelled MSCs demonstrate MSCs lodge in the pulmonary vascular bed where they remain detectable for a few days with the majority of cells being cleared within 24–48 h although this may be prolonged in injured lungs [71, 72]. While in the pulmonary capillary bed, MSCs appear to ‘sense’ the surrounding inflammatory environment through cell surface damage and pathogen molecular pattern receptors [73–75] releasing a range of soluble elements of its ‘secretome’ in response.

MSC homing to sites of injury is mediated via chemokine receptors, adhesion proteins, and matrix metalloproteinase (MMPs) [76]. At injury sites, MSCs interact with target cells through cell-to-cell contact and paracrine/endocrine effects, the latter by secreting soluble mediators (including anti-inflammatory cytokines, antimicrobial peptides, and angiogenic growth factors) and extracellular vesicles (EVs), and/or by transferring organelles such as mitochondria to target immune cells (leukocytes [monocytes, macrophages, lymphocytes] and structural cells [including endothelial, epithelial, and smooth-muscle cells]) [77]. MSC-derived EVs contain proteins, lipids, mRNA, microRNAs, DNAs [78]. Mitochondrial transfer from MSCs can occur through tunnelling nanotubes (TNTs), gap junctions, or via EVs [1, 78–80].

Systemically administered MSCs may also undergo rapid tissue factor-mediated apoptosis in a phenomenon designated the ‘instant blood-mediated inflammatory response’ (IBMIR) [81]. The response of the host immune system to these dead or dying MSCs driving potential beneficial responses for the underlying lung injury [82]. This may be particularly relevant in both non-viral and viral-induced ARDS in the context of increasing recognition of different inflammatory ARDS phenotypes [83].

### Microenvironmental responsiveness and MSC pre-activation

MSCs respond to microenvironmental cues as demonstrated by their release of anti-inflammatory mediators when placed in an inflammatory lung environment [84]. This is mediated by MSC response to damage and pathogen-associated molecular patterns (DAMPs and PAMPs, respectively) [85]. MSC toll-like receptors (TLRs) 3 and 9 are activated by viral RNA and viral unmethylated CpG-DNA, respectively, leading to activation of downstream signalling pathways [2]. Recent reports have characterized MSCs responsiveness to the in vivo inflammatory lung environment by exposing them to either clinical bronchoalveolar lavage fluid (BALF) or serum samples from patients with ARDS [85–89]. Exposure of MSCs to a healthy lung environment (i.e., BALF obtained from healthy volunteers) induced expression of genes encoding for recognition as foreign to the host immune system and for inflammation [85]. In contrast, MSCs exposed to BALF samples from patients with cystic fibrosis or ARDS demonstrated disease-specific responses in gene and protein expression and in downstream effects on immune effector cells such as alveolar macrophages [85, 87, 89].

Pre-activation or ‘licencing’ of MSCs may enhance or direct their therapeutic potential by pre-exposure to conditions that mimic specific microenvironmental conditions [74, 85], or via approaches such as genetic modification [90].

### Direct versus indirect MSC effects

Direct MSC effects may be mediated via cell–cell contact or via its secretome [91]. MSC soluble and insoluble extracellular products can be utilized in place of a whole cell therapy [92], facilitating delivery directly into the lung via nebulization [93, 94]. MSC-secreted angiopoietin-1 (Ang-1) and keratinocyte growth factor (KGF) enhance restoration of disrupted alveolar–capillary barrier, while specific regulatory mRNAs in EVs mediate the protective effects of MSCs in pre-clinical models of bacterial or non-infectious acute lung injuries [95, 96].

## MSCs—insights from pre-clinical studies

There is a large body of literature demonstrating efficacy of either systemic or direct intratracheal (IT) MSC administration in pre-clinical models of acute pneumonia/pneumonitis and acute lung injury. Most of these studies involve acute lung injury induced by bacteria or bacterial products (endotoxin) or other means [97]. The models include both rodents, as well as large animals (pig, sheep) and explanted human lungs [98]. A range of approaches have been utilized for dose size, dosing route, and MSC source. In contrast, there are a relatively small number of pre-clinical studies investigating effects of MSC administration in pre-clinical models of respiratory virus infections.

### MSC effects in viral pneumonia

Systemic MSC administration may reduce the chemokines responsible for lung leukocyte infiltration, such as granulocyte–macrophage colony-stimulating factor (GM-CSF), monocyte chemoattractant protein-1 (MCP-1), and macrophage inflammatory protein-1 alpha (MIP-1α). Antiviral immune responses include increased levels of IFN-γ, which alone or together with pro-inflammatory cytokines activate MSCs to release anti-inflammatory mediators [99]. In vitro, MSCs suppress lymphocyte proliferation in response to the activation of influenza-specific T cells and inhibit the cytotoxicity of specific T cells against H1N1 influenza virus [100]. MSCs may also promote lung epithelial and endothelial cell repair, which may be associated with the anti-inflammatory, anti-apoptotic, and anti-oxidative effects of MSCs, thus promoting endothelial–epithelial barrier integrity, which helps the surfactant to recover followed by a decrease in alveolar oedema and atelectasis [101].

### Strain-dependent efficacy in influenza models

MSC administration improved dysregulated alveolar fluid clearance and protein permeability induced by H5N1 and H7N9 influenza viruses in in vitro models, in part by releasing soluble mediators including Ang-1 and KGF that up-regulated sodium and chloride transporters [102]. In parallel studies, systemic administration of human bone marrow-derived MSCs 5 days after induction of H5N1 infection in aged, immunocompetent mice reduced virus-induced mortality, weight loss, lung oedema, BALF CD4^+^ T cells and natural killer (NK) cells, lung histopathological lesions, pro-inflammatory cytokines and chemokines in the absence of reducing lung virus titres. Notably, no effects were observed in mortality and body weight loss in young mice. These observations suggest that systemic MSC administration may provide benefit in older patients who are at higher risk for severe pulmonary illness caused by H5N1 influenza and also possibly SARS-CoV-2 infection.

Avian influenza virus infection can trigger a very intense pro-inflammatory response compared to other influenza viruses. Avian Influenza virus (H9N2) infection increases serum and lung levels of GM-CSF, MCP-1, MIP-1α and inflammatory leukocyte chemoattractants. In a pre-clinical model of H9N2-induced lung injury using young immunocompetent mice, a single systemic MSC administration (10^5^ MSCs) 3 days after injury resulted in reduction in mortality, lung oedema, histologic injury, BALF and serum chemokines and cytokines (including MCP-1 and MIP-1a), and improved gas-exchange and increased anti-inflammatory mediator concentrations [103]. In another study, systemic administration of UC-MSCs were more effective than BM-MSCs when administered 5 days after induction of Influenza A (H5N1) infection in young female immunocompetent mice with respect to decreasing body weight loss, lung oedema, and inflammation [104]. However, neither type of MSC improved mortality or decreased virus titre. Nonetheless, this has provided one platform for use of UC-MSCs in large number of clinical investigations in COVID-19-induced ARDS.

Despite the potential for MSCs to be effective in the resolution of viral pneumonia, two studies found MSCs not to be protective against influenza respiratory infections in mice. In one, the effects of a single systemic administration in immunocompetent mice of either murine or xenogeneic human bone marrow-derived MSCs were assessed in lung injury induced by mouse-adapted H1N1 or swine-origin pandemic H1N1 [105]. Neither MSC administration, either alone or as an adjuvant therapy with oseltamivir, was effective either when administered prior to virus inoculation, or when therapeutically administered. In another study, neither systemic nor IT administration of 2 doses of MSCs (5 × 10^5^ cells) improved H1N1-mediated lung injury [106].

### MSCs in pre-clinical COVID-19

There are no pre-clinical data investigating effects of MSC administration in models of coronavirus respiratory infection, mostly due to the lack of an established animal model. SARS-CoV-2 replication was observed in several non-human primates and in inbred strains of mice following intranasal infection, but these models failed to show clinical signs of pulmonary disease as seen in humans [107]. Human ACE2-overexpressing transgenic mice infected with SARS-CoV-2 demonstrated interstitial pneumonia with lymphocyte and monocyte infiltration into the alveolar interstitium and accumulation of macrophages in alveolar spaces [108]. While these models require further evaluation, they may facilitate the testing of therapeutics including cell-based therapies for COVID-19. We can appreciate the promise of MSCs for the treatment of COVID-19 infection by taking what we know from pre-clinical studies using infectious reagents of bacterial and viral origin.

### Cell-free therapies

MSCs-derived EVs have been demonstrated to have comparable and in some cases more effective than MSCs themselves in ameliorating inflammation and injury in a range of pre-clinical lung injury models [109, 110]. Systemic administration of porcine MSC-EVs was found to be safe and reduced virus shedding in nasal swabs, influenza replication in the lungs, BALF pro-inflammatory cytokines and chemokines, histopathological changes when administered 12 h after viral inoculation in a mixed swine (H3N2, H1N1) and avian (H9N5, H7N2) influenza-induced pig lung injury model [111]. These findings suggest systemic EV administration has therapeutic potential for respiratory virus-induced lung injuries.

### Summary

There is a substantial pre-clinical literature examining the potential for MSCs to reduce lung injury, via effects that address injury mechanisms directly relevant to viral pneumonia. However, the data from pre-clinical studies of respiratory virus infections are limited to influenza viruses and have produced conflicting results. MSC-derived cell products, particularly EVs, demonstrate significant therapeutic promise.

## MSCs—insights from clinical studies

### MSC safety profile in clinical trials

A recent systematic review and meta-analysis of MSC therapy included 55 randomized control trials with 2696 patients [112] across a wide variety of clinical conditions. Other than an increased risk of pyrexia, no safety signals (including in relation to infection, thromboembolic events or malignancy) were reported. Furthermore, the risk of death was significantly lower in the MSC-treated group compared to controls. Further follow-up data are needed to determine the safety of MSCs in the longer term.

### Clinical trials in ARDS and sepsis

Several ongoing and completed trials of MSCs for ARDS and sepsis support the investigation of MSCs in patients with viral pneumonia. These studies demonstrate that MSC therapy in critically ill ARDS and sepsis patients is feasible and safe, albeit the numbers treated to date is small (see detailed review [113]). Two phase 2 studies in early ARDS demonstrate that MSCs were well tolerated [87, 114]. Post hoc analyses suggested patients with more severe impairment of oxygenation had a better treatment response [114] and that higher MSC viability was associated with greater improvements in Ang-2 and oxygenation [122]. Intravenous MSC therapy reduced lung permeability injury and lung injury severity versus placebo, decreasing mediators of lung injury, such as angiopoietin-2 [115].

### MSCs in viral pneumonia

An observational study investigating the use of menstrual blood-derived MSCs in 17 patients with H7N9 viral-induced moderate-to-severe ARDS reported no safety issues [116]. Patients received a dose of 1 × 10^6^ cells/kg MSCs on multiple (up to 4) occasions in early versus later phase ARDS. MSC therapy in COVID-19 patients has been reported in multiple small uncontrolled studies. There was significant heterogeneity in the population receiving MSCs as well as the regimens used in terms of dose and frequency of administration. In general, these studies reported no safety issues and promising efficacy data, however findings must be considered exploratory due to the many methodological limitations. Randomized controlled clinical trials of MSC therapy for COVID-19 are now being reported (Table 3). The dose used was variable and several of these trials tested multiple doses. Together these trials indicate that MSCs were well tolerated and showed promising efficacy in COVID-19 infection  ([117–119] and NCT04371393). Other trials have recently completed recruitment and results are awaited [120]. Further larger trials are required to adequately assess the question of efficacy of MSCs in viral pneumonia.

**Table 3:** Randomized controlled clinical trials of MSC therapy for COVID-19

Study type/patient cohort	Intervention	Outcomes measured	Reference/trial number
Phase 2 Severe COVID-19 induced ARDS (n = 100, 2:1 ratio)	UC-MSCs (VCANBIO) 4 × 10^7^ MSCs × 3 infusions	Improvement in whole lung lesion volume, no difference in SAEs	Shi et al. [117] NCT04288102)
Phase 1/2a Mild–moderate and moderate–severe COVID-19 induced ARDS (n = 24, 1:1)	UC-MSCs + Heparin 1 × 10^8^ MSCs × 2 infusions	No infusion associated AEs or SAEs, inflammatory cytokines decreased, improved patient survival, and time to recovery	Lanzoni et al. [118] NCT04355728
Phase 1 Critically ill COVID-19 patients (n = 40, 1:1)	UC-MSCs + standard care 1 × 10^6^ MSC/kg	Improved survival rate, no changes in ICU stay or ventilator use, no AEs reported. IL-6 reduced	Dilogo et al. [119] NCT04457609
Phase 3 Moderate-to-severe COVID-19 induced ARDS (n = 223, 1:1)	BM-MSCs (Remestemcel-L) 2 × 10^6^ MSC/kg × 2 infusions	30-day all-cause mortality, ventilator-free days, adverse events, 7-day mortality, ARDS resolution	NCT04371393 Ongoing
Phase 1/2 Moderate-to-severe COVID-19 induced ARDS (n = 120, 1:1)	UC-MSCs (Orbcell-C) Max tolerated dose established in Phase 1	Oxygenation Index, SAE incidence	NCT03042143 Ongoing

## Future directions: opportunities and challenges

While allogeneic MSCs demonstrate considerable therapeutic promise for severe viral pneumonia significant knowledge gaps and challenges remain (Table 4). Translation of MSCs to effective therapy in patients with severe viral pneumonia will require additional studies to determine: (1) the optimal MSCs therapeutic; (2) the optimal dose regimen; (3) the optimal patient population, and (5) the potential interaction of concomitant therapies.

**Table 4:** Challenges for testing mesenchymal stromal cells for ARDS

Challenge	Solutions/options
Source and production methods	Bone marrow, umbilical cord, iPSC-derived
Optimal dose (intravenous)	2, 4, or 10 × 10^6^/kg (ideal body weight)
Number of doses and timing	One dose versus two doses Dose spacing 36–72 h apart?
Inclusion criteria	High-flow nasal oxygen versus invasive mechanical ventilation
Identifying treatment responsive phenotypes	Potential variables—age, viral, bacterial pneumonia, shock or not shock, biological variables (IL-8, Protein C, bicarbonate)

### Optimal MSC therapeutic

Allogeneic MSCs can be harvested from bone marrow, umbilical cord, and adipose tissue, and each of these MSC sources have been tested in small ARDS trials that reported no safety issues [121, 122]. MSCs derived from older donors may have impaired function [123]. Recently, MSCs that were produced with  induced pluripotent stem cell (iPSC) technology were tested in a sheep model of severe pneumonia and sepsis over 48 h; based on a preliminary report, *i-*MSCs markedly improved oxygenation and they reduced protein rich lung lymph flow indicating reduced lung vascular injury compared to placebo controls [124]. There is also pre-clinical work indicating that EVs derived from MSCs can deliver most of the biological cargo from MSCs and might be adapted for clinical treatment, currently being developed for therapy of premature infants with neonatal respiratory distress syndrome [125]. It is not possible at this time to determine the relative efficacy of the various sources of MSCs, although there is considerable interest in iPSC-derived MSCs as they might accelerate and simplify production providing there are no safety issues that emerge in pre-clinical studies, especially regarding oncogenic risk.

### Optimal MSC dosing regimen

The optimal dosing regimen of MSCs for viral pneumonia is uncertain, and pre-clinical studies in small animals provide limited insights regarding dosing or timing of MSC therapy in the clinical setting. In pre-clinical studies, a dose-dependent effect, with greater efficacy seen with higher doses, has been frequently reported [126]. However, in a human LPS model, dose-dependent adverse effects were demonstrated at the highest dose investigated with enhanced febrile response and coagulation activation reported [127]. Furthermore, in a human study of diabetic nephropathy, 150 but not 300 million MSCs improved renal function [128]. Neither lower nor higher doses may be optimal [129]. The dose used in phase 1 and 2a trials [121, 122], and an ongoing 2b clinical trial in ARDS (https://stattrial.com; password—StemCells4All NCT03818854) has been based on a study in a sheep bacterial pneumonia and sepsis model indicating that 10 × 10^6^ MSC/kg was superior to 5 × 10^6^ MSC/kg for reducing pulmonary oedema [130]. Other trials have used lower doses ranging from 2–4-10 × 10^6^ MSCs/kg [114]. More studies will be needed to establish optimal dosing which may also depend on the source of the MSCs and their viability and potency. It is also possible that a higher dose may be needed for ARDS if the patient’s clinical course includes evidence of systemic injury with shock.

Frequency of MSC administration is a further consideration. Although studies in ARDS and sepsis have used a single intravenous infusion of MSCs, data emerging from studies in COVID-19 support efficacy with multiple doses. There is also some pre-clinical evidence to support multiple dosing regiments. A preliminary report in bacterial pneumonia and sepsis in sheep that showed efficacy of two intravenous doses of 10 × 10^6^ i-MSCs/kg spaced 24 h apart [124]. The new evidence that MSCs can reduce biologic evidence of lung injury in BALF 48 h after intravenous administration of 10 × 10^6^ MSCs/kg suggests that perhaps a second dose could be administered between 48 and 72 h to maximize efficacy, especially if the patient was not clinically improving by oxygenation and other pulmonary and systemic criteria.

A further challenge related to dosing regimens is that differing MSC products may not have consistent efficacy. A potency assay to standardize therapeutic efficacy of a dose would be an important development to allow direct comparison of MSC products, however at present this is not available.

### Optimal patient population

The immunomodulatory effect of MSCs raises the potential that they may be more likely to exert therapeutic effects in the subgroup of patients with a dysfunctional pro-inflammatory response to viral infection. Proof-of-concept for this approach comes from research into patients with ARDS, where hypo- and hyper-inflammatory endotypes have been identified [131, 132] that respond differently to therapeutic interventions [133]. Calfee and colleagues reported that a parsimonious set of three biologic markers identified patients with higher mortality versus lower mortality for classical ARDS prior to COVID-19 (interleukin-8, Protein C, and bicarbonate), and this approach worked very well even in a re-analysis of the START-2 trial 60 patient trial of MSCs versus placebo [134]. Other approaches could include focusing on viral pneumonia patients with the highest mortality risk, which would include patients in shock requiring vasopressors. Another approach would be to use a radiographic severity score showing more pulmonary oedema with a RALE score greater than 20 for example, since higher RALE scores is an independent marker of higher mortality [135].

### Impairment of MSCs by viral infection

A potential concern is that viruses may directly infect and impair MSC function due to their expression of receptors which allow the entry of several types of viruses. However, because MSCs are negative for the aACE2 and TMPRSS2 proteins, they are not susceptible to SARS-CoV-2 [136]. Therefore, MSCs would be safe and effective for treating patients with COVID-19 pneumonia. CD147 is another entry receptor for SARS-CoV-2, expressed by tissue-specific stem cells [137] and certain pulmonary cells. Viral infection by either route and intracellular replication results in both loss of airway epithelial cells and regenerating stem cells, thus diminishing cellular and lung regeneration.

### MSCs use with concomitant therapy

One final challenge relates to the potential interaction of concomitant therapies, particularly in the setting of COVID-19 pneumonia. Treatment with steroids is now standard of care for hospitalized patients with COVID-19 requiring oxygen therapy. However, there are data to suggest that steroids may attenuate the beneficial effects of MSCs which will have implications for the impact of MSCs in this setting [138].

## Summary and conclusions

Mesenchymal stem cells display considerable promise for the treatment of more severe viral pneumonia, display potentially relevant mechanisms of action, and have a demonstrated safety profile in early phase studies, MSCS are currently being evaluated in multiple larger phase 2 clinical trials for COVID-19 pneumonia. Important insights will emerge from these studies in the coming months. Nevertheless, important knowledge gaps remain to be elucidated, including the optimal cell type, dose regimen and dose timing, as well as the optimal patient subpopulations for these therapies. There are also challenges to the scale-up of MSC production to conduct large phase 3 studies and for their eventual clinical use should they prove effective for the treatment of viral pneumonia. Further research aimed at optimizing the therapeutic potential of these MSCs for viral pneumonia will continue to be an important priority if we are to realise their therapeutic potential for this devastating condition.

## Supplementary Information

**Additional file 1. Supplementary Table 1**. Antiviral Medications Under investigation for treatment of Influenza.

## Data Availability

Not applicable.
