# Supplementary material for: Mesenchymal stem/stromal cell-based therapies for severe viral pneumonia: therapeutic potential and challenges
Source: Intensive Care Med Exp. 2021 Dec 31;9:61. doi: 10.1186/s40635-021-00424-5 (PMC8718182; doi:10.1186/s40635-021-00424-5)
Supplement: Supplementary file 1 — Additional file 1. Supplementary Table 1. Antiviral Medications Under investigation for treatment of Influenza. [file 40635_2021_424_MOESM1_ESM.docx]

| **Supplementary Table 1. Antiviral Medications Under investigation for treatment of Influenza** | | | | |
| --- | --- | --- | --- | --- |
| **Antiviral Agent** | **Activity Against Virus** | **Development Stage** | **Safety and efficacy** | **Evidence** |
| **CC-42344** | Viral Polymerase 2 inhibitor; inhibits viral replication | Pre-Clinical;  Phase 1 influenza study planned (no reports available) | Results are pending | [1]; [2] |
| **Diltiazem** | Unclear mechanism of action | NCT03212716 – Phase 2 – Severe influenza patients;  Pre-clinical Influenza mouse models | Reductio in viral load and symptoms | [3] |
| **EDP-938** | non-fusion replication inhibitor | NCT03384823 – Phase 1 Safety Healthy volunteer; NCT03691623 – Phase 2a – RSV virus challenge model | Results pending | [4] |
| **EIDD-1931/EIDD-2801** | ribonucleoside analog inhibitor w | Pre-clinical non-human primates, mouse and ferret Influenza models | Reductio in viral load both Influenza A and B | [5] |
| **Eritoran** | Structural analogue of TLR4, blocks interaction with MD2. Blocks release of HMGB1 | Pre-clinical - Influenza mouse models | Reductio in viral load and symptoms | [6]; [7] |
| **Favipiravir** | Polymerase complex inhibitor | NCT02026349 - Phase 3 - influenza patients | Did not significantly improve viral clearance | [8]; [9] |
| **HA minibinders** | High affinity binding to viral haemaglutinin, necessary for viral cell entry | Pre-clinical - Intranasal delivery - Mice and Ferrets | Reduction in viral particles in vitro | [2] |
| **Inhaled GM-CSF (Sargramostim)** | Promotes viral clearance and recovery from injury | NCT02601365 - Phase 1 - influenza patients | Safe - efficacy results pending | [10]. [11]; [12]. |
| **Inhaled Nitric Oxide** | Presumed anti-inflammatory activity | NCT04606407 - Open Label - viral pneumonia patients | Safe - efficacy results pending | [13] |
| **JNJ-53718678** | Fusion protein inhibitor | NCT03379675 – Phase 2a - RSV infected volunteers;  NCT02387606 - Phase 2a - RSV virus challenge model. | Inhibits established acute lower respiratory tract infection | [4];[14]; [15]; [16] |
| **JNJ-64417184** | Non-nucleoside polymerase inhibitor | NCT03403348 – Phase 1 - First-in-Human - healthy volunteer studies | Results are pending | [2] |
| **lumicitabine (ALS-008176)** | RNA Polymerase inhibitor | NCT02673476 – Phase 2a – RSV Patients;  NCT02094365 – Phase 2a – RSV viral challenge model; NCT02202356 – Phase 1 – RSV Infant patients; NCT02478333 – Phase 1 – Healthy subjects;  NCT03333317 - Phase 2 - Infants/Children with RSV | Poor Results in discontinued from development for treatment of RSV | [17]; [18]; [19]. |
| **MHAA4549A** | human immunoglobulin G1 (IgG1) monoclonal antibody to the highly conserved epitope on the stalk of influenza A HA | NCT02623322 – Phase 2 – Uncomplicated Influenza A  NCT02293863 - Phase 2 – Severe Influenza A Patients | Conflicting results | [20]; [21]; [22] |
| **PC786** | Nonnucleoside L protein polymerase inhibitor | NCT03382431 – Phase 1/2 – RSV virus challenge model;  NCT03236233 – Phase 1 – mild asthma subjects;  NCT03715023 – Phase 2 – RSV infection in HSC transplant patients | Significant anti-viral effect against RSV | [23] |
| **Pimodivir (JNJ-63623872)** | PB2 cap-binding inhibitor | NCT03381196 – Phase 3 – early Influenza A patients;  NCT02888327 – Phase 1 – Healthy Volunteers;  NCT03834376 – Pre-approval access – Influenza A Patients;  NCT02659735 – Phase 1 – healthy volunteers;  NCT02532283 - Phase 2 - Patients with influenza A | Conflicting results | [24]; [25]; [26]; [27] |
| **presatovir (GS-5806)** | fusion inhibitor interrupts entrance of virus into cells | NCT01797419 – Phase 1 – RSV patients <24 months;  NCT01756482 – Phase 2 – RSV virus challenge model;  NCT02254421 – Phase 2 - RSV infection in HSC transplant patients | Safe but did not improve virologic or clinical outcomes versus placebo | [28]; [29]; [30] |
| **Quercetin** | naturally occurring dietary flavonoid interfere with various stages of virus entry and replication cycle | Pre-Clinical - Phase 1 study planned - Patients infected with Influenza (no reports available) | Conflicting results | [31];[32]; [33] |
| **RSV M2-1 Inhibitors** | Respiratory Syncitial Virus M2-1 V transcription anti-termination factor inhibitor | Pre-clinical - In vitro and in silico models | enhances viral clearance and reduces inflammation | [34] |
| **Umifenovir (Arabidol)** | fusion inhibitors; it interacts with the virus hemagglutinin and thus prevents fusion of the viral envelope with cell membranes. | NCT01651663 – Phase 4 - Treatment and Prophylaxis of Influenza and Common Cold;  NCT03787459 – Phase 3 – Severe Influenza patients | Reduction of time to resolution of all symptoms | [35] |
| **VH244** | Therapeutic Interfering Particle -mutant interferes with viral replication, genomic interference and enhancement of the innate immune response | Pre-clinical (Prophylactic mouse and ferret models) | Improved outcomes and enhanced viral clearance | [2]; [36] |
| **VIS410** | monoclonal antibody to influenza A virus, in combination with baloxavir and neuraminidase inhibitors | NCT02468115 - Phase 2a – Influenza A (H1N1) virus challenge model | Reduces symptoms | [37] |
| **Ziresovir (AK0529)** | fusion inhibitor interrupts entrance of virus into cells | NCT03699202 – Phase 2 – RSV infected adults;  NCT02297594 – Phase 1 – Healthy subjects;  NCT03400995 – Phase 1 – Healthy male subjects;  NCT02460016 – Phase 1 – RSV infant patients;  NCT04231968 – Phase 3 – RSV infant patients | No published data in humans or animals | [38]; [39] |

**References cited in Table**

1. Cocrystal Pharma, I. *CC-42344 - Influenza A PB2 Inhibitor*. 2021; Available from: <https://www.cocrystalpharma.com/development-pipeline/influenza/cc-42344>.

2. Beigel, J.H., et al., *Advances in respiratory virus therapeutics - A meeting report from the 6th isirv Antiviral Group conference.* Antiviral Res, 2019. **167**: p. 45-67.

3. Pizzorno, A., et al., *In vitro evaluation of antiviral activity of single and combined repurposable drugs against SARS-CoV-2.* Antiviral Res, 2020. **181**: p. 104878.

4. Rhodin, M.H.J., et al., *EDP-938, a novel nucleoprotein inhibitor of respiratory syncytial virus, demonstrates potent antiviral activities in vitro and in a non-human primate model.* PLoS Pathog, 2021. **17**(3): p. e1009428.

5. Toots, M., et al., *Characterization of orally efficacious influenza drug with high resistance barrier in ferrets and human airway epithelia.* Sci Transl Med, 2019. **11**(515).

6. Perrin-Cocon, L., et al., *TLR4 antagonist FP7 inhibits LPS-induced cytokine production and glycolytic reprogramming in dendritic cells, and protects mice from lethal influenza infection.* Sci Rep, 2017. **7**: p. 40791.

7. Patel, M.C., et al., *Serum High-Mobility-Group Box 1 as a Biomarker and a Therapeutic Target during Respiratory Virus Infections.* mBio, 2018. **9**(2).

8. Wang, Y., et al., *Phase 2a, open-label, dose-escalating, multi-center pharmacokinetic study of favipiravir (T-705) in combination with oseltamivir in patients with severe influenza.* EBioMedicine, 2020. **62**: p. 103125.

9. Doi, Y., et al., *A Prospective, Randomized, Open-Label Trial of Early versus Late Favipiravir Therapy in Hospitalized Patients with COVID-19.* Antimicrob Agents Chemother, 2020. **64**(12).

10. Herold, S., et al., *Inhaled granulocyte/macrophage colony-stimulating factor as treatment of pneumonia-associated acute respiratory distress syndrome.* Am J Respir Crit Care Med, 2014. **189**(5): p. 609-11.

11. Halstead, E.S., et al., *GM-CSF overexpression after influenza a virus infection prevents mortality and moderates M1-like airway monocyte/macrophage polarization.* Respir Res, 2018. **19**(1): p. 3.

12. Paine, R., 3rd, et al., *A randomized trial of recombinant human granulocyte-macrophage colony stimulating factor for patients with acute lung injury.* Crit Care Med, 2012. **40**(1): p. 90-7.

13. Garren., M., Ashcraft, M, Qian, Y, Douglass, M, Brisbois, EJ, Handa, H, *Nitric oxide and viral infection: Recent developments in antiviral therapies and platforms.* Appl Mater Today, 2021. **22:100887**.

14. de la Loge, C., et al., *Monitoring Severity of Respiratory Syncytial Virus (RSV) in Infants and Young Children Using the Pediatric RSV Electronic Severity and Outcome Rating System (PRESORS): Results of Initial Quantitative Validation.* Patient Relat Outcome Meas, 2021. **12**: p. 247-265.

15. Roymans, D., et al., *Therapeutic efficacy of a respiratory syncytial virus fusion inhibitor.* Nat Commun, 2017. **8**(1): p. 167.

16. Stevens, M., et al., *Antiviral Activity of Oral JNJ-53718678 in Healthy Adult Volunteers Challenged With Respiratory Syncytial Virus: A Placebo-Controlled Study.* J Infect Dis, 2018. **218**(5): p. 748-756.

17. Patel, K., et al., *Respiratory syncytial virus-A dynamics and the effects of lumicitabine, a nucleoside viral replication inhibitor, in experimentally infected humans.* J Antimicrob Chemother, 2019. **74**(2): p. 442-452.

18. Brendish, N.J. and T.W. Clark, *Antiviral treatment of severe non-influenza respiratory virus infection.* Curr Opin Infect Dis, 2017. **30**(6): p. 573-578.

19. Wang, G., et al., *Discovery of 4'-chloromethyl-2'-deoxy-3',5'-di-O-isobutyryl-2'-fluorocytidine (ALS-8176), a first-in-class RSV polymerase inhibitor for treatment of human respiratory syncytial virus infection.* J Med Chem, 2015. **58**(4): p. 1862-78.

20. Deng, R., et al., *Pharmacokinetics of the Monoclonal Antibody MHAA4549A Administered in Combination With Oseltamivir in Patients Hospitalized With Severe Influenza A Infection.* J Clin Pharmacol, 2020. **60**(11): p. 1509-1518.

21. Deng, R., et al., *Pharmacokinetics of MHAA4549A, an Anti-Influenza A Monoclonal Antibody, in Healthy Subjects Challenged with Influenza A Virus in a Phase IIa Randomized Trial.* Clin Pharmacokinet, 2018. **57**(3): p. 367-377.

22. Lim, J.J., et al., *A Phase 2 Randomized, Double-Blind, Placebo-Controlled Trial of MHAA4549A, a Monoclonal Antibody, plus Oseltamivir in Patients Hospitalized with Severe Influenza A Virus Infection.* Antimicrob Agents Chemother, 2020. **64**(7).

23. DeVincenzo, J., et al., *Safety and Anti-viral Effects of Nebulized PC786 in a Respiratory Syncytial Virus Challenge Study.* J Infect Dis, 2020.

24. Finberg, R.W., et al., *Phase 2b Study of Pimodivir (JNJ-63623872) as Monotherapy or in Combination With Oseltamivir for Treatment of Acute Uncomplicated Seasonal Influenza A: TOPAZ Trial.* J Infect Dis, 2019. **219**(7): p. 1026-1034.

25. McKimm-Breschkin, J.L., et al., *Prevention and treatment of respiratory viral infections: Presentations on antivirals, traditional therapies and host-directed interventions at the 5th ISIRV Antiviral Group conference.* Antiviral Res, 2018. **149**: p. 118-142.

26. O'Neil, B., et al., *A Phase 2 Study of Pimodivir (JNJ-63623872) in Combination with Oseltamivir in Elderly and NonElderly Adults Hospitalized with Influenza A Infection: OPAL study.* J Infect Dis, 2020.

27. Trevejo, J.M., et al., *Pimodivir treatment in adult volunteers experimentally inoculated with live influenza virus: a Phase IIa, randomized, double-blind, placebo-controlled study.* Antivir Ther, 2018. **23**(4): p. 335-344.

28. Marty, F.M., et al., *A Phase 2b, Randomized, Double-blind, Placebo-Controlled Multicenter Study Evaluating Antiviral Effects, Pharmacokinetics, Safety, and Tolerability of Presatovir in Hematopoietic Cell Transplant Recipients with Respiratory Syncytial Virus Infection of the Lower Respiratory Tract.* Clin Infect Dis, 2020. **71**(11): p. 2787-2795.

29. Stray, K., et al., *Drug Resistance Assessment Following Administration of Respiratory Syncytial Virus (RSV) Fusion Inhibitor Presatovir to Participants Experimentally Infected With RSV.* J Infect Dis, 2020. **222**(9): p. 1468-1477.

30. Porter, D.P., et al., *Assessment of Drug Resistance during Phase 2b Clinical Trials of Presatovir in Adults Naturally Infected with Respiratory Syncytial Virus.* Antimicrob Agents Chemother, 2020. **64**(9).

31. Mehrbod, P., et al., *Quercetin as a Natural Therapeutic Candidate for the Treatment of Influenza Virus.* Biomolecules, 2020. **11**(1).

32. Uchide, N. and H. Toyoda, *Antioxidant therapy as a potential approach to severe influenza-associated complications.* Molecules, 2011. **16**(3): p. 2032-52.

33. Cocrystal Pharma, I. *Cocrystal Pharma Completes IND-enabling Studies with CC-42344 for the Treatment of Seasonal and Pandemic Influenza A, Plans to initiate a Phase 1 Trial in the Third Quarter*. 2021 [cited 2021 17 September]; Available from: <https://www.globenewswire.com/news-release/2021/06/23/2251702/0/en/Cocrystal-Pharma-Completes-IND-enabling-Studies-with-CC-42344-for-the-Treatment-of-Seasonal-and-Pandemic-Influenza-A-Plans-to-initiate-a-Phase-1-Trial-in-the-Third-Quarter.html>.

34. Wang, L., et al., *Discovery of a Novel Respiratory Syncytial Virus Replication Inhibitor.* Antimicrob Agents Chemother, 2021. **65**(6).

35. Pshenichnaya, N.Y., et al., *Clinical efficacy of umifenovir in influenza and ARVI (study ARBITR).* Ter Arkh, 2019. **91**(3): p. 56-63.

36. Biotherapeutics, V. *Virion Biotherapeutics Demonstrates Preclinical Proof-of-Concept for First Therapeutic Interfering Particle Candidate in Respiratory Virus Infections*. 2018 [cited 2021; Available from: <https://www.businesswire.com/news/home/20181114005102/en/Virion-Biotherapeutics-Demonstrates-Preclinical-Proof-of-Concept-for-First-Therapeutic-Interfering-Particle-Candidate-in-Respiratory-Virus-Infections>.

37. Sloan, S.E., et al., *Clinical and virological responses to a broad-spectrum human monoclonal antibody in an influenza virus challenge study.* Antiviral Res, 2020. **184**: p. 104763.

38. Gao, Y., et al., *Evaluation of Small Molecule Combinations against Respiratory Syncytial Virus In Vitro.* Molecules, 2021. **26**(9).

39. Zheng, X., et al., *Discovery of Ziresovir as a Potent, Selective, and Orally Bioavailable Respiratory Syncytial Virus Fusion Protein Inhibitor.* J Med Chem, 2019. **62**(13): p. 6003-6014.
